# The Future of British Nursing

**Published:** 1916-08-12

**Authors:** 


### THE MATRONS' AND SISTERS' DEPARTMENT.

### THE FUTURE OF BRITISH NURSING.

### I. NURSES AND CENTRAL EXAMINATIONS.

(*Communicated.*)

It may be stated without fear of contradiction that, as regards organisation and centralisation, the development of the profession of nursing has been slow. Whatever may have been the causes conducing to it, the fact remains that nursing education has been left, decentralised, subject to the views and even the whims of hospital and infirmary committees and boards of management, each working on separate lines and approximating in method only as the result of very limited consultation, of imitation, and of the experience gained elsewhere by individual members. There is probably to-day no other great educational system in which students are taught, examined, and turned out as qualified to practise under conditions which can fairly be regarded as haphazard, confused, often unfair to the probationers and misleading to the public.

The would-be nurse has hitherto often had to seek a portal to her profession with inadequate information. Having, by the aid of the excellent manuals published for her guidance and the advice of friends, distinguished between a "general training" and the instruction and experience obtainable in a special or fever hospital, a cottage hospital, or an asylum, she has had no authoritative standard by which to judge of the quality of the education she will receive or of its value to her in her subsequent career. She has had to choose her training-school, and, having chosen it, for good or evil to abide by it and by its management, which she gradually finds is beyond outside control. It may be argued that a system can be judged only by its results, and that the British system of nursing training stands out as unique in its triumphant success throughout the world. It has certainly been at its best wonderfully successful, as the result of the keen and untiring interest and the spirit of emulation shown by the teaching staffs of the most efficient training-schools. It is in no spirit of disparagement that the demands for some further protection for the nurse and the nursing profession, and for "recognition," have arisen. A standard of recognised training has been adopted by the Local Government Board, and the registration of nurses, which is in its simplest form merely the recognition of a standard of knowledge and ability, has been widely demanded. The College of Nursing movement aims not only at establishing registration, but at securing ultimately for the nursing profession similar advantages and responsibilities to those secured for the medical profession by the General Medical Council, the Royal Colleges, and other examining bodies.

The General Nursing Council which it is proposed to set up is to control registration (including the authority of removal from the Register, subject to certain powers of appeal) and the conditions of admission to the Register, including nurse training, *examination, the appointment of examiners*, and the formation of local boards. The effect of the clauses concerning examinations and the appointment of examiners undoubtedly will be, and is intended to be, the establishment of central examinations for nurses, and, as a necessary consequence, the standardisation of the training for the pass examination for qualification to register. Hardly sufficient stress has so far been laid upon this inevitable sequence of the passing of the Registration Act.

Undoubtedly the time has come for central qualifying examinations in nursing. It is an anomaly and an anachronism that a nurse should receive her certificate of three years' training after no examination other than that held by the authorities of her local training-school with the assistance of an "independent" examiner selected and appointed by themselves. Some tentative attempts at central examinations have actually been made; such, for instance, as the appointment at several training-schools of the same "outside" examiner. A proposal to hold central examinations for nurses trained at Poor-Law infirmary training-schools was discussed some years ago; fortunately this suggestion, which might have differentiated Poor-Law nurses and been detrimental to their interests from the point of view of public opinon, was not carried out, and the Poor-Law institutions are free to present their nurses at central examinations side by side with those trained in the general hospitals.

That both training-schools and nurses are fully prepared for and anticipate the coming of central examinations we fully believe. It would be a most unfair and unfounded suggestion of lack of adaptability on the part of the women of to-day to suppose otherwise. Nurses are already accustomed to the principle of central examination; strangely enough, whilst the all-important certificate of three years' nursing training is granted after training and private examination at all sorts of institutions for the sick, central examinations are, and have long been held by the Medico-Psychological Association, formerly by the Obstetrical Society, and now by the Central Midwives Board and by the Incorporated Society of Trained Masseuses, the examinations of the last-named body being sufficiently searching to necessitate much study and application. Nurses are no longer regarded as either too ill-educated or too sensitive to face an examiner; they need not nowadays be submitted in class for *viva-voce* examination (a method which we know to have been actually adopted in the past) in order to spare them from facing the examiner alone!

Central examinations will be for the benefit of the nurse and of the nursing profession. The training-school and its curriculum, submitted to a public comparison of results, and subject to some supervision by an established authority, will be kept up to the mark. The nurse will no longer be tied, restricted, or influenced by the idiosyncrasies or limitations of her training-school and its examiners. The requirements of preparation for central examination will have to be met by the establishment of a definite curriculum, as the result of which a levelling up of the present standards of teaching may be confidently expected. In the issue of The Hospital for August 5 last we called attention to the subjects of instruction set out for probationers at King's College Hospital: many such a synopsis is excellent; few, if any, are complete or beyond criticism; all may be improved under the influence of standardisation. We look with confidence for an extension of the excellent system of instruction preliminary to ward-training, of the teaching of sick-cookery and domestic economy, and of those special branches of nursing work now too often neglected in individual schools.

An innovation is only too often regarded as a criticism and a hardship. We are convinced, however, that the nurse will be the gainer by the introduction of the central examination and one portal method of admission to her profession, and that registration is by no means the only, and perhaps not the greatest, of the ends which will follow the incorporation of enactment of the Nurses' Registration Act and the College of Nursing.

